# Self-Practice of Stabilizing and Guided Imagery Techniques for Traumatized Refugees via Digital Audio Files: Qualitative Study

**DOI:** 10.2196/17906

**Published:** 2020-09-23

**Authors:** Catharina Zehetmair, Ede Nagy, Carla Leetz, Anna Cranz, David Kindermann, Luise Reddemann, Christoph Nikendei

**Affiliations:** 1 Department of General Internal Medicine and Psychosomatics Center for Psychosocial Medicine University Hospital of Heidelberg Heidelberg Germany; 2 Department of Clinical Psychology, Psychotherapy and Psychoanalysis University of Klagenfurt Klagenfurt Austria

**Keywords:** stabilizing techniques, guided imagery, refugees, qualitative analyses, posttraumatic stress disorder, mental health, PTSD, audio, therapy

## Abstract

**Background:**

Refugees have an increased risk of developing mental health problems. There are insufficient psychosocial care structures to meet the resulting need for support. Stabilizing and guided imagery techniques have shown promising results in increasing traumatized refugees’ emotional stabilization. If delivered via audio files, the techniques can be practiced autonomously and independent of time, space, and human resources or stable treatment settings.

**Objective:**

This study aimed to evaluate the self-practice of stabilizing and guided imagery techniques via digital audio files for traumatized refugees living in a reception and registration center in Germany.

**Methods:**

From May 2018 to February 2019, 42 traumatized refugees participated in our study. At T1, patients received digital audio files in English, French, Arabic, Farsi, Turkish, or Serbian for self-practice. Nine days later, at T2, a face-to-face interview was conducted. Two months after T2, a follow-up interview took place via telephone.

**Results:**

At T2, about half of the patients reported the daily practice of stabilizing and guided imagery techniques. At follow-up, the average frequency of practice was once weekly or more for those experiencing worse symptoms. No technical difficulties were reported. According to T2 and follow-up statements, the techniques helped the patients dealing with arousal, concentration, sleep, mood, thoughts, empowerment, and tension. The guided imagery technique “The Inner Safe Place” was the most popular. Self-practice was impeded by postmigratory distress factors, like overcrowded accommodations.

**Conclusions:**

The results show that self-practice of stabilizing and guided imagery techniques via digital audio files was helpful to and well accepted by the assessed refugees. Even though postmigratory distress factors hampered self-practice, “The Inner Safe Place” technique was particularly well received. Overall, the self-practiced audio-based stabilizing and guided imagery techniques showed promising results among the highly vulnerable group of newly arrived traumatized refugees.

## Introduction

With a prevalence rate of approximately 40%, mental health is a major problem for refugees in their host country [1]. Posttraumatic stress disorder (PTSD) is one of the most commonly reported mental health issues [1,2]. Multiple studies support trauma exposure approaches like narrative exposure therapy, trauma-focused cognitive behavioral therapy (CBT), and eye movement desensitization and reprocessing to address PTSD symptoms in the refugee population [1,3,4]. However, displaced people face many obstacles limiting preconditions for trauma exposure therapy, such as frequent reallocation, uncertainty regarding their asylum application outcome as well as financial, intercultural, and language barriers upon arrival [5]. Furthermore, a premature trauma confrontation should be avoided [6]. Hence, providing traumatized refugees with initial stabilizing treatment may be very helpful until the refugees’ surroundings are sufficiently stable for trauma confronting treatment approaches.

Evidence is growing that stabilization techniques positively affect refugees’ mental health [7-9]. In this context, stabilizing and guided imagery techniques in line with Reddemann [10] are promising treatment approaches for adult and minor refugees with PTSD in individual and group therapy approaches [11-14]. The use of stabilizing and guided imagery techniques seems helpful in securing an initial emotional stabilization even under unstable conditions [13,14]. However, all face-to-face therapeutic interventions with refugees face problems of language heterogeneity and the need for space, time, and human resources. Given the fact that online self-help interventions are almost as effective as face-to-face interventions [15,16], modern media with app- and web-based alternatives offer possibilities to bridge these barriers impeding traditional face-to-face psychotherapy.

Web- and app-based studies in health-related areas have increased significantly in recent years. Systematic reviews show positive results from web- and app-based interventions [17-21]. Especially for patients with trauma-related disorders, web-based CBT [22-28], coping strategy programs [29,30], as well as computer games to re-consolidate traumatic intrusive memories [31,32], have been shown to have promising effects in reducing trauma symptoms [33]. Further, app-based self-help interventions using image- and audio-based formats show encouraging effects on distress, PTSD, and depression symptoms [16,34-37]. Therefore, digital psychosocial interventions are a promising approach for asylum seekers and refugees.

This study builds on earlier studies using stabilizing and guided imagery techniques in newly arrived refugees [13,14] to evaluate traumatized refugees’ self-practice of stabilizing and guided imagery techniques via digital audio files using in-depth qualitative analysis. Our research questions were the following: (1) What kind of practicing behavior is shown? (2) Which technical difficulties are reported? (3) What clinical effects do the patients achieve through stabilizing and guided imagery techniques via digital audio files? (4) What difficulties are reported in the self-practice of the techniques via audio files? In previous studies, the guided imagery technique called “The Inner Safe Place” has played a key role [11,12], yet little is known about the experiences with this technique. Hence, we have additionally focused on the question of (5) experiences specific to the guided imagery technique, “The Inner Safe Place.”

## Methods

### Participants and Study Design

We conducted a prospective, descriptive study using qualitative semistructured interviews. The setting was the refugee state registration and reception center “Patrick Henry Village” (PHV), Heidelberg-Kirchheim, Germany. At the PHV, the University Hospital of Heidelberg, in cooperation with physicians in private practice, operates a medical and psychosocial walk-in clinic [38,39]. Between the end of May and early December 2018, refugees who sought help in the psychosocial walk-in clinic [38] and met our study’s inclusion criteria were referred to treatment with the audio-based stabilizing and guided imagery techniques. Due to follow-up interviews conducted two months later, the overall study period (recruitment and follow-up) was from May 2018 until February 2019. Inclusion criteria were a diagnosis of PTSD, access to a personal smartphone for digital audio file delivery, and fluency in speaking and understanding of one of the following languages: English, French, Arabic, Farsi, Turkish, or Serbian. Exclusion criteria were substance addiction, current psychosis, and age under 18 years. All participants had applied for asylum in Germany or were in the process of applying during the intervention.

### Procedure and Ethics

**Figure 1:**
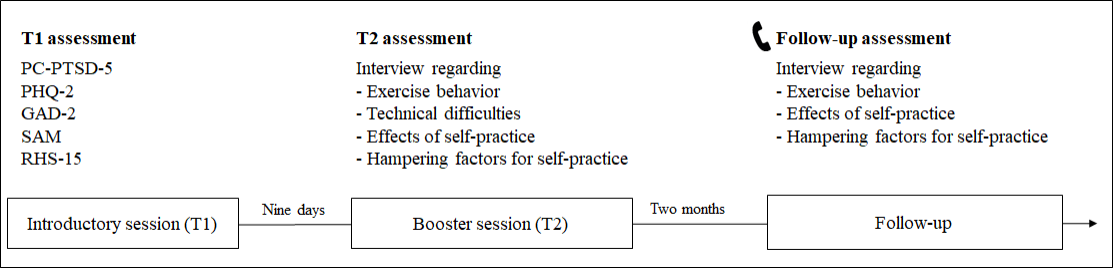
An overview of the study procedure.

When indicated, the psychiatrists and psychotherapists of the psychosocial walk-in clinic recommended treatment with the audio-based stabilizing and guided imagery techniques to patients and made a face-to-face appointment for an introductory session. During the introductory session (T1), the patients first were asked to complete a baseline measurement via a tablet. Afterward, the psychologist discussed psychoeducational issues with the patients and informed them of the content of the audio files and the aim of stabilizing and guided imagery techniques. Then, the psychologist and the patients practiced the stabilizing and guided imagery techniques together once using the audio files and subsequently discussed the effects and difficulties of each technique. Finally, the audio file was transferred to the patient’s phone in the appropriate language. The introductory session lasted approximately two hours. The booster session (T2) took place nine days later and particularly focused on counseling and feedback regarding difficulties in practicing the techniques using audio files. Since the focus of this study was put on the self-practice of stabilizing and guided imagery techniques using the provided audio files, no further guided practice sessions were undertaken. An interview was conducted with each patient at the end of the session. Two months after T2, the patients were contacted again by phone and interviewed for a second time (follow-up). Three attempts were made to reach the patients for follow-up interviews. If necessary, a telephone or face-to-face translator was involved during the introductory session and the booster session. Figure 1 provides an overview of the study procedure.

The study was approved by the ethics committee of the University of Heidelberg (S-640/2016), and all participants gave their written informed consent in accordance with the Declaration of Helsinki.

### Audio Files

The audio file consisted of three parts, namely (1) mindful breathing, (2) the body scan, and (3) the guided imagery technique “The Inner Safe Place,” which are described in more detail elsewhere [13,14]. The audio files were available in English, Arabic, Farsi, French, Turkish, and Serbian (spoken by about 80% of the refugee population in the PHV). Before, the text material of the techniques had been translated by professional interpreters and translators into the respective language and then digitally recorded by native speakers. Except for the Arabic version, all versions were narrated by a female native speaker. The audio files and written instructions are available free of charge [40]. If desired, a printed instruction booklet is also available for a nominal fee.

### Psychometric Baseline Assessment (T1)

Prior to the introductory session (T1), a baseline measurement including the Primary Care PTSD Screen for DSM-5 (PC-PTSD-5) [41], the two-item Patient Health Questionnaire (PHQ-2) [42], the short version of the General Anxiety Disorder questionnaire (GAD-2) [43], the Self-Assessment Manikin scale (SAM) [44], and the distress thermometer of the Refugee Health Screener-15 (RHS-15 distress thermometer) [45] were used to assess participants’ mental distress.

The PC-PTSD-5 [41] assesses PTSD symptoms via a list of different trauma events and five binary questions (0 = “no” and 1 = “yes”) on re-experiencing, avoidance, physical reactions, emotional numbness, and trauma-distorted feelings of guilt and blame [41]. With a total score between 0 and 5, individuals with a score ≥3 are identified as patients with probable PTSD. The PC-PTSD-5 shows good sensitivity (.93), acceptable specificity (.85), and good acceptance by patients [46].

The PHQ-2 [42] assesses major depression via two items on anhedonia and depressed mood. Both items are rated on a scale of 0 (not at all) to 3 (nearly every day) and give a total score of 0 to 6. The PHQ-2 shows good construct validity (r from 0.67-0.87), good internal consistency (α=.83) [47]. An overall cut-off score at ≥3 provides a sensitivity of .61 to.87 and specificity of .86 to .92 for major depression in primary care and medical outpatients [42,47,48].

The GAD-2 [43] assesses anxiety disorders via two items on anxiousness and worrying. The GAD-2 ranges from 0 (not at all) to 3 (nearly every day) with a cut-off score of ≥3. The GAD-2 shows a sensitivity of .89 and specificity of .83 for generalized anxiety disorder [43,49]. Internal consistency reliability is acceptable (α=.83) [50].

The SAM [44] is a nonverbal, cross-cultural rating scale [51] widely used for diverse groups of patients, such as for traumatized patients or refugees [52-55]. The individual can choose between five manikin pictures representing the present affective state on the dimensions valence (sad to happy), arousal (exited to calm), and dominance (weak to strong) [53].

The RHS-15 [45] was developed specifically for refugees and asylum seekers. It comprises 14 symptom items that can be answered on a 5-point Likert-Scale. A “distress thermometer” assesses perceived distress on a visual analog scale ranging from 0 to 10. The RHS-15 authors define a screening as positive with a cut-off score set at ≥12 for the symptom items or ≥5 if the distress thermometer is used. The RHS-15 distress thermometer can be used independently and has a good sensitivity (.81-.95) and specificity (.86-.89) [45].

### Semistructured Qualitative Interviews

We used qualitative, semistructured interviews to obtain data illuminating the participants’ experiences with the audio files and audio-based stabilizing and guided imagery techniques. The semistructured interviews comprised key questions, which were followed by probing questions; for further details, clarifying questions could be added. Multimedia Appendix 1 shows the interview guidelines and key questions.

### Quantitative and Qualitative Data Analysis

Quantitative statistical analysis was carried out by using the Statistical Package for the Social Sciences program version 24 [56]. Demographic variables and baseline characteristics were analyzed using descriptive statistics (frequencies, means, and standard deviations). The qualitative interviews were digitally recorded and transcribed verbatim by independent co-workers using the guidelines for interview transcription presented in Mayring [57]. Statements regarding the refugees’ practicing behavior were analyzed descriptively. The other research questions were analyzed with the software MAXQDA [58] following the principles of inductive content analysis described by Mayring [57]. Here, sentences are identified as the most basic units of meaning [59], summarized into relevant categories, and further grouped into main themes. The categories and main themes were subsequently discussed to reach consensus or to be adjusted if required [57]. The T2 and follow-up statements have been summarized to facilitate presentation; noteworthy differences between T2 and follow-up interviews are explicitly highlighted.

## Results

### Sample Characteristics

A total of 83 patients attending the psychosocial walk-in clinic in PHV were referred to treatment with audio-based stabilizing and guided imagery techniques. Of these, 42 patients (50%) attended the introductory session (T1). All patients attending the introductory session consented to participate. They were aged between 19 and 51 years (mean 33.67, SD 8.3). All patients were invited to attend a booster session nine days later. The booster session (T2) was attended by 19 of 42 patients (45%). Reasons for non-attendance were reallocation (n=7, 17%), self-initiated departure from PHV (n=4, 10%), conflicting appointments (n=2, 5%), as well as reported as inactive (n=1, 2%), hospital stay (n=1, 2%), and imprisonment (n=1, 2%). We do not know why the remaining 7 (17%) patients did not attend. For the follow-up interviews, we attempted to contact as many of the 42 patients as possible. We were unable to reach 18 patients (43%) owing to inactive or incorrect phone numbers, while 7 (17%) patients were not successfully contacted after three attempts. In total, we conducted follow-up interviews with 19 of the participating patients (45%). Table 1 shows the sample characteristics for the total sample of 42 patients attending at least the first session.

**Table 1:** Sociodemographic characteristics and measurement at baseline for all participants (N=42).

Characteristic			Value
**Gender, n (%)**			
	Male		25 (60)
	Female		17 (40)
**Region of origin, n (%)**			
	Middle East		23 (55)
	Balkan Peninsula		3 (7)
	North Africa		4 (9)
	Sub-Sahara Africa		12 (29)
**Medication, n (%)**			
	None		10 (24)
	Antidepressant		27 (64)
	Neuroleptics		3 (7)
	No information		2 (5)
**Religion, n (%)**			
	Christianity		10 (24)
	Islam		30 (72)
	Judaism		1 (2)
	Other		1 (2)
**Questionnaire scores, mean (SD)**			
	Primary Care PTSD^a^ Screen for DSM-5^b^		3.93 (0.84)
	PHQ-2^c^		3.87 (1.57)
	GAD-2^d^		4.12 (1.56)
	**SAM^e^**		
		Valence	4.05 (1.10)
		Arousal	2.79 (1.60)
		Dominance	2.98 (1.12)
	RHS-15^f^ thermometer		7.21 (2.22)

^a^PTSD: posttraumatic stress disorder.

^b^DSM-5: Diagnostic and Statistical Manual of Mental Disorders, 5th Edition.

^c^PHQ-2: Two-item Patient Health Questionnaire.

^d^GAD-2: General Anxiety Disorder questionnaire.

^e^SAM: Self-Assessment Manikin scale.

^f^RHS-15: Refugee Health Sreener-15.

### Psychometric Baseline Assessment (T1)

The baseline scores for PTSD, depression, anxiety disorders, perceived distress, and affective state are presented in Table 1. On average, the patients reported four trauma experiences (mean 4.02, SD 1.71, range 1-7). The most frequently reported traumatic events were experiencing torture (27/42, 64%), being physically or sexually assaulted or abused (25/42, 60%), being imprisoned (23/42, 55%), seeing someone being killed or seriously injured (23/42, 55%), experiencing a war (20/42, 48%), and losing a loved one through homicide or suicide (16/42, 38%). Except for one patient, all patients confirmed three trauma symptoms and fulfilled the criteria of a possible PTSD according to the PC-PTSD-5. Furthermore, 28 (66%) patients reported suffering from four to five different PTSD symptoms. Additionally, 30 patients (71%) fulfilled the criteria of major depression, and 32 patients (76%) displayed symptoms of a generalized anxiety disorder. Furthermore, 38 patients (90%) scored positive on emotional distress assessed by the RHS-15 distress thermometer.

### Descriptive Results of Practice Behavior Revealed by Interviews

Table 2 depicts the results of the practice behavior reported at T2 and follow-up.

**Table 2:** Statements regarding the self-practice behavior of stabilizing and guided imagery techniques.

Variables			T2^a^	Follow-up^b^
**Frequency of practice, n (%)**				
		2-3/day	4 (21)	4 (21)
		1/day	6 (31)	0 (0)
		2-4/week	5 (26)	6 (31)
		1/week	2 (11)	5 (26)
		Stopped	2 (11)	4 (21)
**Techniques were experienced as, n (%)**				
		Helpful	14 (74)	11 (58)
		Partly helpful	3 (16)	7 (37)
		Not helpful	2 (11)	1 (5)
**Most helpful technique,** **n (%)**				
		Breathing	4 (21)	8 (42)
		Body Scan	1 (5)	0 (0)
		Guided imagery	10 (53)	8 (42)
		No statement	4 (21)	3 (16)
**Place for self-practice^c,d^, n (%)**				
	Room		14 (82)	14 (93)
	Outside		5 (29)	4 (27)
	No statement		3 (18)	0 (0)
**Time of self-practice^c,d^, n (%)**				
	Morning		9 (53)	4 (27)
	Daytime		9 (53	3 (20)
	Evening		8 (47)	9 (60)
	If symptoms were perceived		0 (0)	6 (40)
	No statement		4 (24)	2 (13)

^a^N=19 patients, who attended the booster session (T2), interviews held face to face directly after the booster session.

^b^N=19 patients who were available via telephone two months after the booster session (follow-up), interviews conducted via telephone.

^c^These sections only include the answers of patients practicing the techniques: T2 n=17, follow-up n=15.

^d^Multiple answers were possible.

### Results of the Inductive Content Analysis of the Interviews (T2 and Follow-Up)

We identified and coded 344 single statements from the T2 interviews and 334 single statements from the follow-up interviews. From these codes, we created twelve categories, which were then summarized into four main themes. Examples of each category are shown in Multimedia Appendix 2.

#### The Audio File as a Tool for Self-Practice (68 Codes)

In the T2 interviews, the patients gave feedback regarding the audio files at the technical and content levels.

##### Technical Difficulties With the Audio Files (38 Codes)

Most patients (n=15, 79%) denied any technical difficulties with the audio files, while 4 (21%) patients reported technical difficulties largely in connection with their smartphone. One patient could not listen to the audio file because his smartphone was stolen, and another’s smartphone broke. One patient reported difficulties in retrieving the audio file on his smartphone. The fourth patient received the audio file via e-mail because his smartphone was unable to connect with the other device for transferring the audio file in the introductory face-to-face session.

##### Structure of the Audio Files (30 Codes)

Most of the patients stated that the voice and speed of the speech felt comfortable and natural for them. Some patients shared that they memorized the instructions. One patient remarked that the entire audio file might be too long as he sometimes falls asleep before the audio file has finished. Another appreciated the audio files but said it was not comparable to face-to-face contact with a therapist. Furthermore, some patients said that they were unable to follow the instruction of closing their eyes as it caused them discomfort.

#### Effects of Audio-Based Stabilizing and Guided Imagery Techniques (187 Codes)

The patients described stabilizing and guided imagery techniques via audio files as valuable for the relief of several clinical symptoms.

##### Arousal (44 Codes)

Most of the patients stated that stabilizing and guided imagery techniques helped them to feel more relaxed and calm immediately after practicing as well as in the long-term. One patient reported being able to regulate his heartbeat better. Others felt more comfortable with practice, both mentally and physically. Some patients noticed that they felt less stressed in the long run, which motivated them to continue practicing.

##### Tension and Sleep (38 Codes)

Most of the patients stated that stabilizing and guided imagery techniques helped them to reduce body tension immediately after practice. Some felt less pain in the lower back or neck. One patient shared that the techniques helped her with breathing because the tension and stress around her neck had improved. The patients further stated improved sleep during the night and a reduction in feelings of hardly ever getting a good night’s sleep or merely a few hours of sleep. In follow-up interviews, some patients highlighted feeling as if they had more physical energy for their everyday lives.

##### Thoughts and Concentration (50 Codes)

At T2, most of the patients stated that the techniques helped them to focus on the here and now. Ten patients mentioned improvements in their concentration in the short- and long-term, such as when going to German language classes. Some mentioned feeling less forgetful than before or remembering more things than before. Some patients shared that they were able to escape from the problems of their everyday life while practicing the techniques. At follow-up, they frequently mentioned that they had fewer negative thoughts, fewer worries, and more positive thoughts compared to before practicing the techniques.

##### Mood and Empowerment (55 Codes)

Most patients stated feeling pleasure and relief during self-practice. Some reported feeling better while practicing the techniques, and others reported being in a good mood after practicing. One patient said that he felt increased inner freedom during the practice of the techniques; others felt renewed hope. Additionally, some patients reported feeling revitalized after practicing at follow-up. Three patients mentioned that they were able to get closer to and communicate better with other people. One participant said that he started going into town and engaging in small talk. One patient also reported feeling greater confidence.

#### Difficulties With the Audio-Based Stabilizing and Guided Imagery Techniques (110 Codes)

Patients reported internal and external challenges with self-practice of the stabilizing and guided imagery techniques.

##### Accommodation Situation (40 Codes)

The patients struggled with the living conditions in their current accommodation and reported feeling very uncomfortable. For most patients, it was difficult to cohabit with so many different people. Many patients said there was no space for them to calm down and use the techniques. If they were a family, the entire family shared one room without time and space to oneself; some shared with their children and had to take care of them. Many reported difficulties because of the very noisy environment on top of the lack of privacy.

##### Lack of Concentration (34 Codes)

The patients said it was difficult for them to concentrate on the instructions and remain focused. Several patients mentioned that recurring and painful thoughts, like worries about family members in their home country, the asylum procedure, or fear of persecution, would distract them. Others remarked that memories of bad events or periods sometimes came to mind during self-practice. Some patients stated they were so depressed and withdrawn that they did not want to practice or listen to anything.

##### Only Short-Term Relief (36 Codes)

Several patients indicated difficulties in accepting that the relief from the stabilizing and guided imaging techniques was only temporary. Some of the patients stated that, at the outset, they had too-high expectations of the long-lasting effect of the techniques. One patient reported feeling disappointed because he still felt burdened by symptoms despite having practiced the techniques. The patients mentioned that they sometimes felt that the techniques did not affect their well-being because they were unable to combat the problems they faced every day.

#### Appraisal of the Guided Imagery Technique ‘The Inner Safe Place” (71 Codes)

Containing statements about positive and negative effects during practice as well as on its content, the guided imagery technique “The Inner Safe Place” was a central aspect within the participants’ statements.

##### Positive Effects of the Guided Imagery Technique (23 Codes)

The participants said that they appreciated the guided technique “The Inner Safe Place.” They described this technique as most helpful, liked, particularly positive, and stabilizing as it occasionally allowed them to be far away from their worries during practice. They felt a sense of freedom and security during this technique. One patient reported that he still felt safe three hours after having practiced “The Inner Safe Place.” Another said he felt he had regained some hope through this technique.

##### Difficulties With the Guided Imagery Technique (32 Codes)

Patients also described various difficulties while practicing “The Inner Safe Place.” The patients reported that they felt so comfortable imagining an inner safe place that they immediately felt burdened or even sad when they returned to reality. Some patients mentioned that it was difficult for them to visualize a place in their mind’s eye. Another patient said that their inner safe place had shattered. The patients found that “The Inner Safe Place” technique could not always protect them from distractions and recurring thoughts during practice.

##### Content Statements Regarding the Guided Imagery Technique (16 Codes)

The patients shared some of their inner safe places with us. Some chose to be alone in their inner safe place, while others imagined having their family or friends there with them. If they had imagined themselves there alone, they usually thought of places in nature, like a beach, the ocean, or grassy plains. One patient said that he thought of Germany for his inner safe place, while another patient reported seeing himself cooking in a kitchen. Three patients reported imagining themselves either in their future or back in their childhood.

## Discussion

### Principal Findings

This study aimed to investigate the self-practice of stabilizing and guided imagery techniques via digital audio files in newly arrived refugees living in a state registration and reception center. The qualitative results show that the self-practice of audio-based stabilizing and guided imagery techniques can help traumatized individuals experience symptom relief in the early stages of arrival in their host country. Although some difficulties in practicing the exercise were reported, “The Inner Safe Place” was perceived as the most helpful technique in delivering positive inner images and feelings. However, self-practice of audio-based stabilizing and guided imagery techniques require a high degree of self-motivation and commitment from affected refugees. Finding such motivation and commitment may sometimes be overtaxing in light of impeding internal factors, such as lack of concentration, or postmigratory stressors, like lacking privacy in the accommodations.

Concerning aspects of the user application, the patients’ statements indicate that using audio files is feasible and practicable. Issues only arose because of device problems. Various studies have described that the majority of refugees own a smartphone, yet not everyone in this group has access to the internet [60]. In the current study, the audio file was transferred to the patient’s smartphone via USB cable or Bluetooth. The MP3 format appears to be a robust and simple format available offline for everyone. In a study by Zehetmair et al [14] assessing stabilizing and guided imagery techniques in a group setting, participating refugees voiced a desire for instructions to facilitate practicing the techniques between the face-to-face group sessions. By developing an audio-based approach in this study, every participant always had the technique instructions with them. The files are available for download [40].

Examining the participants’ practicing behavior, we found that 52% of the patients described the daily (to several times daily) self-practice of the stabilizing and guided imagery techniques at T2. However, the statements indicated a decreasing tendency of self-practice and more flexible use of the audio files at follow-up. On the one hand, this may be explained by the high degree of self-motivation and commitment the self-practice of the techniques requires of the highly burdened patients causing them to abate over time. Furthermore, the booster session could also have been perceived as a monitoring element, so they may have practiced more before T2 than after when they practiced at their own accord without an anchoring/monitoring session. Nevertheless, at follow-up, still 52% of the patients practiced the stabilizing and guided imagery techniques frequently, with statements ranging from daily to several times a week.

The qualitative results of the T2 and follow-up interviews showed that audio-based stabilizing and guided imagery techniques were able to alleviate the symptoms associated with mental stress. The participating refugees described changes in arousal levels, mood and empowerment, thoughts and concentration, as well as sleep and tension. Perceived symptom changes through the practice of stabilizing and guided imagery techniques immediately after practice or over time are consistent with other outcomes [13]. Several studies encourage the use of stabilizing and guided imagery techniques in traumatized patients: for example, increases in conscious action (eg, consciously change the attention focus) and decreases in hyperarousal, emotional numbness, and perceived stress have been shown [61,62]. These changes lead to an experience of improved situation control and self-efficacy [10,61,63]. According to Reddemann [63], the combination of self-calming elements and elements of internal process recognition increases internal stabilization.

Nevertheless, the participants described external and internal impeding factors for the self-practice of stabilizing and guided imagery techniques via audio file. Newly arrived refugees are faced with problems such as frequent reallocation, uncertainties regarding their asylum application, as well as uncertainties about social, cultural, or future-related aspects. Moreover, refugees in refugee camps report inhuman living conditions, forced passivity, and waiting, all of which exacerbate their feelings of a lack of control regarding their current situation [64-66]. Accordingly, the patients in our study reported that the stabilizing and guided imagery techniques provided them with short term symptom relief but were unable to offer any solutions for their overwhelming and stressful everyday life’s problems. Furthermore, our participants were not only preoccupied with worries about the present and future but also described experiencing trauma-related symptoms, which often made it difficult for them to stay focused or engage in the techniques. Although the audio files were designed to promote stabilization and distressing memory distancing, we cannot rule out that participants may be triggered by hearing the language of their home country, which is often strongly associated with trauma. Despite the high burden of traumatic symptoms, patients reported they were able to consciously shift the focus of their thoughts to the techniques.

“The Inner Safe Place” was considered the most helpful technique by the majority of patients. It helped patients experience positive feelings of security, hope, well-being, and freedom. These emotions are particularly meaningful and soothing for traumatized refugees who have been subjected to persecution, imprisonment, and torture [67,68]. Several of them were able to transfer the resulting positive feelings to their present situation. This ability can motivate the refugees to continue practicing the respective techniques. Guided imagery techniques aim at eliciting positive feelings that traumatized individuals often are unable to feel by creating an inner image that contrasts the traumatic re-experience imagery and is available in situations of overwhelming emotions and thoughts [10]. This further can facilitate self-distancing from the traumatic experiences and the associated symptoms of emotional overload and intrusions [69]. However, participants also reported encountering some difficulties with the technique as the positive inner images often clashed harshly with their realities in the present. On the one hand, this highlights that the patients were able to engage in the technique and felt comfortable at their imaginary inner place; on the other hand, it also underpins the effect of post migratory stressors by contrasting their current living situation in a state registration and reception center with the place they had imagined for themselves.

### Limitations

This study has several limitations. First, the study relies on self-reports regarding both the questionnaire assessment as well as the qualitative interviews. Therefore, a tendency towards compliant or socially desirable behavior or answers cannot be ruled out. Nevertheless, we conducted 38 interviews (19 at T2, 19 at follow-up) with n=27 of the total sample of 42 patients participating in the study, which is sufficient to achieve saturation for main themes in heterogeneous populations [70]. Second, the patients were of different ages, education, and country of origin. This heterogeneity limits the generalizability of our results but also reflects the realities of the sample group. Third, the PHV psychosocial walk-in clinic’s psychiatrists and psychotherapists recommended treatment with audio-based stabilizing and guided imagery techniques to *N*=83 refugees and asylum seekers. The study included 42 patients who took part in the study and attended the introductory session (T1), equivalent to a dropout rate of 50%. Unfortunately, we do not have any information about the reasons for non-attendance. However, among other reasons, logistical barriers, like reallocation to different accommodations, scheduling conflicts, missing the appointment, as well as treatment enrollment barriers, such as treatment-related insecurities and fears or symptom- and anticipated outcome-related motivational issues may be possible explanations Additionally, there was a high dropout rate of 55% between T1 and T2. As already experienced in previous studies [13,14,71], the registration and reception center presents a research setting that is inherently afflicted by frequent reallocation and high dropout rates. The high dropout rate limits the generalizability of our results and can further have facilitated the occurrence of the selection bias. Fourth, we cannot neglect that there might have been other circumstances that affected the patients’ symptom load. So, even though the patients attributed symptom changes to the self-practice of the techniques, psychopharmaceutic or other stabilizing effects cannot be ruled out. Nevertheless, the positive effect attribution leads to increased commitment and motivation to continue practicing the techniques. Fifth, the impact of the respective narrators’ voice characteristics (male, female, etc) was not further assessed in this study. However, none of the patients reported difficulties related to the narrator’s voice but rather gave positive feedback regarding the audio file narration during their interviews. However, we cannot rule out that some patients might have encountered problems with the specific voice narrating their audio file.

### Conclusions

This study explored refugees’ perspectives on the self-practice of stabilizing and guided imagery techniques via digital audio files after they arrived in a state registration and reception center. The stabilizing and guided imagery techniques via audio files proved to be a practical and effective tool for self-help regardless of the patient’s country of origin or ethnic background. In sum, the participants reported more positive effects than difficulties with the audio-based stabilizing and guided imagery techniques. They described effects on cognitive, emotional, physical, and empowerment levels; difficulties encountered were associated with internal impeding factors, such as lack of commitment, concentration, and only experiencing short-term relief, or external hindering factors, such as lack of privacy in their accommodations. Particularly the use of “The Inner Safe Place” technique was reported to produce pleasant and self-calming feelings. However, the experience sometimes stood in stark contrast to the patients’ daily reality, making it difficult for some patients to cope with the difference. Overall, the qualitative data presented show promising results for the use of audio-based stabilizing and guided imagery techniques in this sample group.

## Appendix

Multimedia Appendix 1 Interview guideline for interviews conducted after the booster session (T2-interview) and two months after the booster session (Follow-up).

Multimedia Appendix 2 Main themes, their categories, and example codes derived from qualitative inductive content analyses of 38 interviews with 27 participants.

## Abbreviations

CBT: cognitive behavioral therapy
GAD-2: General Anxiety Disorder questionnaire
PC-PTSD-5: Primary Care PTSD Screen for DSM-5
PHQ-2: two-item Patient Health Questionnaire
PHV: Patrick Henry Village
PTSD: posttraumatic stress disorder
RHS-15: Refugee Health Screener-15
SAM: Self-Assessment Manikin scale
