# Supplementary material for: Self-Practice of Stabilizing and Guided Imagery Techniques for Traumatized Refugees via Digital Audio Files: Qualitative Study
Source: J Med Internet Res. 2020 Sep 23;22(9):e17906. doi: 10.2196/17906 (PMC7542415; doi:10.2196/17906)
Supplement: Multimedia Appendix 1 [file jmir_v22i9e17906_app1.docx]

| T2 interview guideline |
| --- |
| - Have you practiced the stabilizing and guided imagery techniques by yourself since the last face-to-face session? |
| - How helpful did you find the techniques? |
| - Which technique was the most helpful for you? |
| - How often did you practice? |
| - Where did you practice the techniques? |
| - How did you find the audio-files? |
| - What difficulties did you face during practicing? |
| - What effects did you perceive during practicing? |
| Follow-up interview guideline |
| - Have you practiced the stabilizing and guided imagery techniques by yourself since the last face-to-face session? |
| - How helpful did you find the techniques? |
| - Which technique was the most helpful for you? |
| - How often did you practice? |
| - Where do you practice the techniques? |
| - What difficulties did you face during practicing? |
| - What effects did you perceive during practicing? |
