# Supplementary material for: Self-Practice of Stabilizing and Guided Imagery Techniques for Traumatized Refugees via Digital Audio Files: Qualitative Study
Source: J Med Internet Res. 2020 Sep 23;22(9):e17906. doi: 10.2196/17906 (PMC7542415; doi:10.2196/17906)
Supplement: Multimedia Appendix 2 [file jmir_v22i9e17906_app2.docx]

| Categories and themes | Codes |
| --- | --- |
| 1. The audio-file as a tool for self-practice |  |
| • Technical difficulties  with the audio-files | *‘No, I didn’t have any technical problems.’* |
|  | *[Yes, the smart phone is broken and she could not play it. And that’s the reason why she could not do the exercises [..] She did not try to practice the exercises because her smart phone was broken]* |
| - Structure of the audio-files | *[So, he could not concentrate, he says that he could internalize 70% without using the smart phone. So, that he knows how the exercises work]* |
|  | *‘Maybe sometimes they help me, maybe sometimes I don’t want to listen to the message. Because I need somebody, I will be with him and he will be teaching me face-to-face and not from the phone.’* |
| 1. Effects of audio-based stabilizing and guided imagery techniques |  |
| • Arousal | *[My daily routine is quite stressful and hectically and, therefore, I really enjoy some ease, to calm down, to relax when I listen to it]* |
| • Tension and sleep | *[She is happier, she has less worries. Her sleep is better. She now sleeps five, six hours and she is very relaxed. She feels much better and she wants to continue. She feels 80% better.]* |
|  | *[He says that while he is perceiving and practicing this audio-file, it also has a direct effect on his posture and he is no longer so tense]* |
| - Thoughts and concentration | *‘I felt a sense of relief, I feel good, I feel relaxed, I feel comfortable. And most of the things in my brain, I can feel good about them and I can try to feel a different side of the situation I’m in. Not to worry too much about what is coming and what is going, about the past and the present, just to have my time, I enjoy it, just to be good.’* |
|  | *[It does not work out for 100%, but he recognizes a lot of changes, positive changes, mental health changes, no physical changes. Currently he goes to school now and recognizes what effect the exercises have on him. He can concentrate much more and does not forget so many things. He recognizes improvements of concentration and calmness in school. And this is something positive, too]* |
| - Mood and empowerment | *[oh yes, in any case I am very motivated now. Now I have the feeling that I want to do everything, to go out, to work. Not the way I was before where I wanted to sleep, crawl into bed, see no one. It's definitely not like that anymore.]* |
|  | *[after the exercises there are no negative effects, it is always only something positive. [...] It always gives him a good mood and also good thoughts, and it also always gives him hope to continue to fight and carry on]* |
| 1. Difficulties with the audio-based stabilizing and guided imagery techniques |  |
| • Accommodation situation | *[So the problem with him is the space that he// He lacks the space where he can calm down. That's why he did not continue practicing, because he// They're in a cramped, big room, but they're all with children and the wife. He cannot concentrate. And he needs rest so that he can do the exercises]* |
| • Lack of concentration | *‘I had problems. Sometimes, maybe I’m listening to the exercise, and before you know, my mind just takes off. Something just comes up in my mind sometimes.’* |
| • Only short-time relief | *‘I used the exercises. It’s helpful but it is for a short time. I remember, back then, I discovered, after the exercises, for a moment, I feel at ease. But there are some things, when it comes, it comes. And the exercise is also there for the temporary, after the exercises, for some time, when I do the exercises, I temporarily feel at ease, for some time.‘* |
| 1. Appraisal of the guided imagery technique ‘The Inner Safe Place” |  |
| • Positive effects of the guided imagery technique | *[He likes the safe place the most. And also that he can relax his body. So he forgets where he is and can concentrate on the safe place.]* |
| • Difficulties with the guided imagery technique | *‘Like, in the exercises, it told me to forget about my worries and everything I am going through, and try to picture myself in this safe place. But I can’t even see the safe place, because right now, I am not even safe. I am not safe. When I just think about this, I feel hopeless.’* |
| • Content statements regarding the guided imagery technique | *[I always dream of sitting more or less in the direction of the river or the sea, where there is always running water. I also hear chirping birds very often. Naturally and that I also play with my feet in the water, back and forth. So I feel the water tickle on my feet ]* |

*Note*. With regard to the exemplarily codes, quotes from English speaking patients were marked as direct quotes with quotation marks ‘ ’; quotes from all other patients were translated and are marked with brackets ( ).
